# Revisiting the Multifaceted Roles of Bacteriocins: The Multifaceted Roles of Bacteriocins

**DOI:** 10.1007/s00248-024-02357-4

**Published:** 2024-02-14

**Authors:** Sara Arbulu, Morten Kjos

**Affiliations:** Faculty of Chemistry, Biotechnology and Food Science, Norwegian University of Life Sciences, Ås, Norway

**Keywords:** Bacteriocin, Microbial competition, Bacterial communication, Biofilm, Horizontal gene transfer, Quorum sensing

## Abstract

Bacteriocins are gene-encoded antimicrobial peptides produced by bacteria. These peptides are heterogeneous in terms of structure, antimicrobial activities, biosynthetic clusters, and regulatory mechanisms. Bacteriocins are widespread in nature and may contribute to microbial diversity due to their capacity to target specific bacteria. Primarily studied as food preservatives and therapeutic agents, their function in natural settings is however less known. This review emphasizes the ecological significance of bacteriocins as multifunctional peptides by exploring bacteriocin distribution, mobility, and their impact on bacterial population dynamics and biofilms.

## Introduction

In the environment, many microbes live in polymicrobial communities occupying ecological niches which may be limited in space and resources [1, 2]. To circumvent these conditions, microbes have developed competition and communication strategies that allow them to survive and coexist with other strains and the surrounding environment [3]. Competition may occur by exploitation when one strain restricts competitors’ access to resources [3], or by interference when an individual causes direct harm to their competitors [3, 4]. Microbe-microbe interactions by interference competition can occur by contact-dependent mechanisms such as secretion systems [5, 6] or by the release of antimicrobial compounds such as bacteriocins that target the opponent cell [2].

Bacteriocins constitute a diverse group of ribosomally synthesized antimicrobial peptides or proteins, that have been described across all bacterial phyla. While bacteriocins have been extensively studied for their potential as food preservatives or antimicrobials [7], the actual role of bacteriocins in bacterial communities is less clear. The conventional hypothesis is that bacteria use bacteriocins to kill neighboring cells in the fight for common resources, but their exact role in different competitive settings in nature remains poorly understood. Bacteriocins may also have additional ecological functions, such as mediating cell lysis to release DNA for natural transformation or interact with regulatory networks in the bacterial cells [8, 9]. Here, we first present an overview of the broad range of structures, biosynthesis pathways, and mechanism of action among bacteriocins, and then we discuss different aspects of bacteriocins in relation to their distribution and impact on the surrounding niche as well as their potential antimicrobial and non-antimicrobial roles.

## The Diverse Nature of Bacteriocins

Bacteriocins were first described in *Escherichia coli* in the early twentieth century [10] and the term quickly embraced the antimicrobial peptides produced by gram-positive bacteria [11–13]. The extended bacteriocin family exhibits remarkable diversity in terms of size, genetic organization, structures, antimicrobial spectrum, and mode of action as outlined in recent comprehensive reviews [14, 15]. In the following paragraphs, we offer a brief glimpse into the diverse nature of bacteriocins to emphasize the breadth of this diversity. Classification of bacteriocins is continuously evolving [16–19]. Here, bacteriocins of gram-negative and gram-positive bacteria are presented separately, but it should be noted that there are examples of similar bacteriocins which can be produced by bacteria across the phylogenetic tree.

**Fig. 1:**
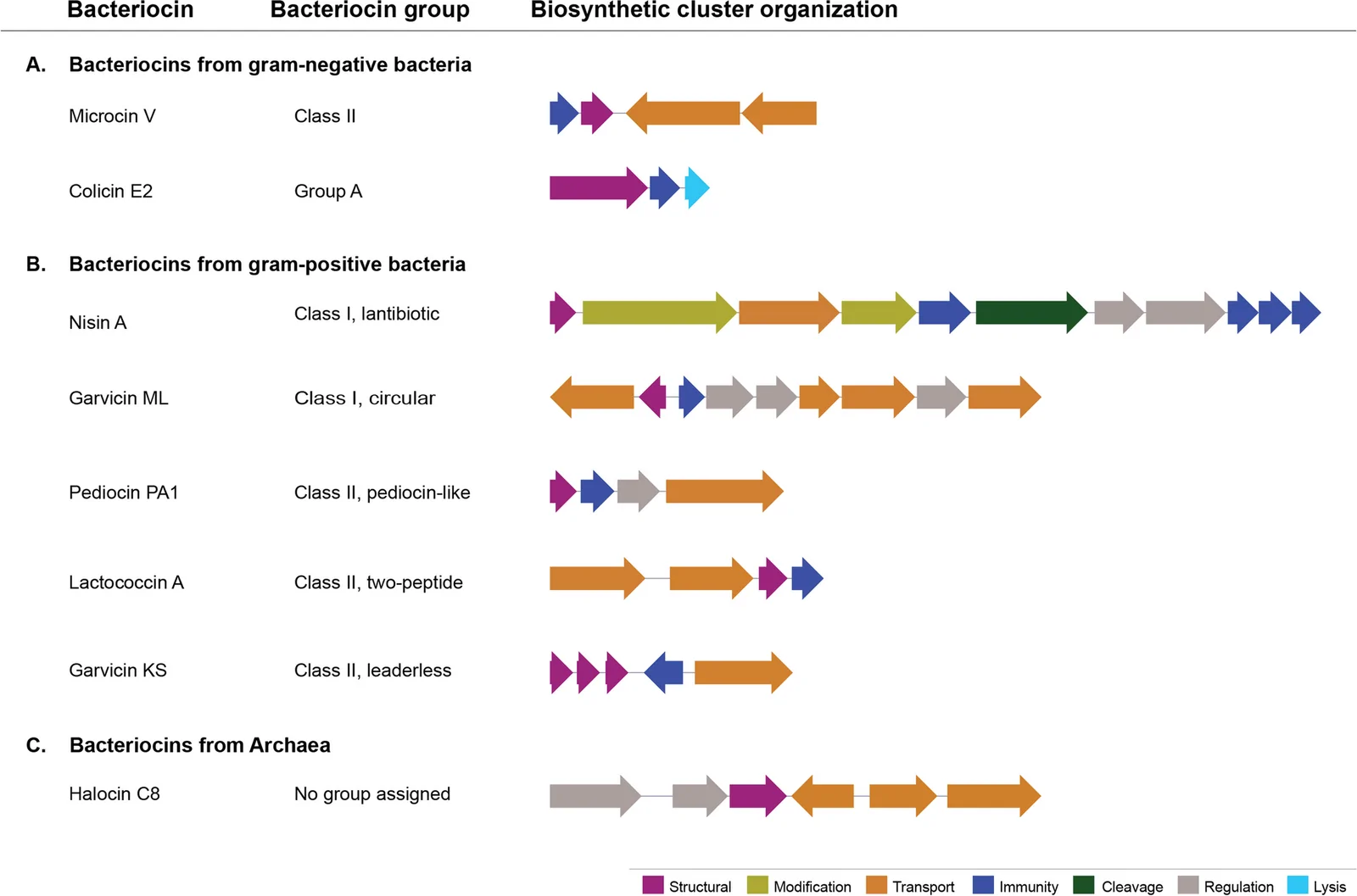
Schematic representation of bacteriocin gene clusters showing the diversity of gene arrangement for bacteriocin synthesis in different bacteriocin groups. Genes are colored according to their function: structural (purple), modification (light green), transport (orange), immunity (dark blue), cleavage (dark green), regulation (grey), and lysis (light blue). References: microcin V [25], colicin E2 [29], nisin A [30], pediocin PA1 [31, 32], lactococcin A [33, 34], garvicin ML [35, 36], garvicin KS [37], halocin C8 [38]. Note that the gene involved in the circularization (modification) of garvicin ML has not been definitely established but likely involves either *gar*X or the *gar*BCDE operon [36]

Among gram-negative bacteria, colicins (30–80 kDa) and microcins (1–10 kDa) from *E*. *coli* have been studied most extensively, although other colicin-like bacteriocins, such as the S-type pyocins produced by *Pseudomonas aeruginosa* and *Klebsiella* spp. [14, 20] have been described. Both colicins and microcins generally have relatively narrow antimicrobial spectra, targeting phylogenetically related bacteria [14, 20]. Colicins exert their antimicrobial function either by pore formation, acting as nucleases, or interfering with the peptidoglycan metabolism. Colicins are encoded on small (group A) or larger (group B) plasmids with a variable genetic organization which usually includes a structural bacteriocin gene, an immunity gene encoding a protein for self-protection, and a gene encoding a lytic protein involved in the release of colicin [21]. On the other hand, microcins can be plasmid or chromosome encoded and are commonly divided into class I (small peptides < 5 kDa), which undergo posttranslational modifications, e.g. microcin J25, microcin B17 or microcin E492 [22–24]; and class II (larger peptides, 5–10 kDa), which include modified and unmodified peptides such as microcin V and microcin H47 [25, 26]. Their biosynthetic gene clusters include a variable number of genes involved in the production, immunity, and export of the peptide [27] (see Fig. 1 for examples of bacteriocin cluster organization). Due to the different modifications, there is a large structural and functional diversity among microcins. Among the most prominent examples are microcin J25, a cyclic peptide containing a C-terminal segment that is threaded through the cycle (thus referred to as a lasso-peptide) which acts as an RNA-polymerase inhibitor; and microcins E492 and H47 which carry a siderophore-like structure on their C-termini needed for the peptides to cross the outer membrane of target cells via recognition by bacterial siderophore receptors to cause membrane depolarization [28]. As exemplified here, the mechanisms of action of microcins and colicins are diverse, and can also include DNase or RNase activity and inhibitors of protein or cell wall synthesis [21], in addition to membrane disruption and transcription inhibition mentioned above.

The study of gram-positive bacteriocins has primarily focused on those produced by lactic acid bacteria. The first classification by Klaenhammer in 1993 [16] was expanded by Nes et al. (1996), then Cotter et al. (2005), and more recently updated by Alvarez-Sieiro et al. (2016) [17–19]. In the latter, three classes are suggested: class I are heat-stable peptides of less than 10 kDa which undergo modifications during their biosynthesis (includes the subgroups lantibiotics, circular bacteriocins, sactibiotics, lasso peptides, and glycosylated bacteriocins); class II are also small heat-stable peptides of less than 10 kDa, but these peptides are not modified post-translationally (includes subgroups like the pediocin-like bacteriocins, the two peptide bacteriocins, leaderless bacteriocins, and other non-modified peptides); lastly class III, are larger (> 10 kDa) heat-labile bacteriocins which include both bacteriolysins and proteins with non-lytic modes of action. Despite most bacteriocins showing a narrow antimicrobial spectrum targeting species related to the producer, some gram-positive bacteriocins such as the canonic lantibiotic nisin show a broader antimicrobial spectrum [39–41].

Like bacteriocins from gram-negative bacteria, bacteriocin class I and II biosynthetic gene clusters include a variable number of genes and operons on the chromosome or on plasmids. These include at least one structural gene, encoding the antimicrobial precursor peptide, and an immunity gene [15]. Most bacteriocins also have a dedicated transporter encoded in the same locus. Intriguingly, the bacteriocin immunity mechanism of some class I and II bacteriocins involves both the immunity protein and a dedicated ABC transporter system, highlighting a complex regulation for self-protection. This is exemplified by the class I lantibiotic nisin [42–44], the circular bacteriocins AS-48 [45–47], or the class II leaderless bacteriocin aureocin A53 [48]. In the case of class I bacteriocins, genes involved in modifying the peptides post-translationally are also present. Finally, regulatory genes for control of production are also often present in these loci, making it possible for the bacteria to adapt the energy costly bacteriocin production to the social environmental conditions [49] (see Fig. 1 one for examples of bacteriocin cluster organization).

Gram-positive bacteriocins of class I and II commonly exert their killing action through cell permeabilization and pore formation that causes the cell to leak essential components and eventually lead to the cell death [50]. However, the mode of action varies between different classes of bacteriocins, and exact mechanisms are mostly uncharacterized. Docking molecules such as lipid II [51–54], the mannose phosphotransferase system (man-PTS) [55, 56] or the intramembrane site-2 protease RseP [57–60] have been shown to be necessary for the function of some gram-positive bacteriocins; however, it is also possible that some peptides can target bacterial membranes without the need for a specific receptor [61].

In addition to the bacteriocin groups mentioned above, archaeal antimicrobial peptides and proteins, known as archaeocins, have been described, including halocins produced by halophilic *Archaea* [62, 63]. Furthermore, there are also other peptides and proteins often referred to as bacteriocins, such as the so-called phage tail-like bacteriocins which have been found across gram-positive (e.g., *Clostridium* spp.) and gram-negative bacteria (e.g., type R- and F-pyocins from *P*. *aeruginosa*, or carotovoricin from (*Erwinia carotovora*) [64–67].

## Bacteriocin Producers are Widespread in Natural Environments

It has been stated that every bacteria is able to produce at least one bacteriocin [16]. Multi-bacteriocinogenic strains have also been frequently described [68–71]. A vast number of studies over the years have explored different environments for the presence of bacteriocin producer strains, including but not limited to recent studies of fermented foods and dairy products [72–75], the human and animal gastrointestinal tracts [76–81], clinical samples [79, 82, 83], medical devices [84], and environmental samples such as the rhizosphere [85] as well as hypersaline [63] or high-temperature habitats [86]. Moreover, the rise of whole genome and metagenomic sequencing in combination with in silico bioinformatic tools for bacteriocin mining such as Bagel4 [87], antiSMASH [88], Bactibase [89], BADASS [90], and BaPreS, [91] are giving a great impulse to the discovery of bacteriocins and bacteriocin-related genes in sequencing data. Bacteriocin-related genes are indeed found in the vast majority of surveyed microbial communities [73, 81, 92–95]. The widespread occurrence of bacteriocins and their associated genes, either complete or incomplete gene clusters, could be the result of evolutionary conservation and habitat adaptation driven by the roles they play in resource competition. However, it may also point to a versatile nature of these peptides in fulfilling multiple functions.

Nevertheless, accurately quantifying the prevalence of bacteriocins and bacteriocin-associated genes in different populations remains challenging. Notably, current wet-lab studies are skewed towards finding pathogen-killing molecules, often overlooking bacteriocins with activity against bacteria less relevant from a clinical point of view. Furthermore, determining the bacteriocin prevalence is also impeded by the diverse range of identification methods employed and the difficulty in mimicking the optimal conditions for bacteriocin production in laboratory settings. Indeed, most of the in silico detected bacteriocin gene clusters are “silent,” indicating low or no expression under laboratory conditions [93, 96–98]. To investigate the potential antimicrobial function of such silent bacteriocin clusters, in silico-identified bacteriocin genes can be revived by heterologous expression, chemical synthesis, or cell-free peptide synthesis systems [96, 99–102]. Nevertheless, the roles of these silent gene clusters in nature often remain obscure. Techniques that can be employed to decipher the expression of silent secondary metabolites under different conditions include using high-throughput elicitor screening with reporter genes to evaluate expression levels and identify inducers [103, 104]. Such approaches, combined with large-scale proteomics from diverse environments, will be critical in the future to get a more holistic view of the prevalence of bacteriocins in natural settings.

## Regulation of Bacteriocin Production

**Fig. 2:**
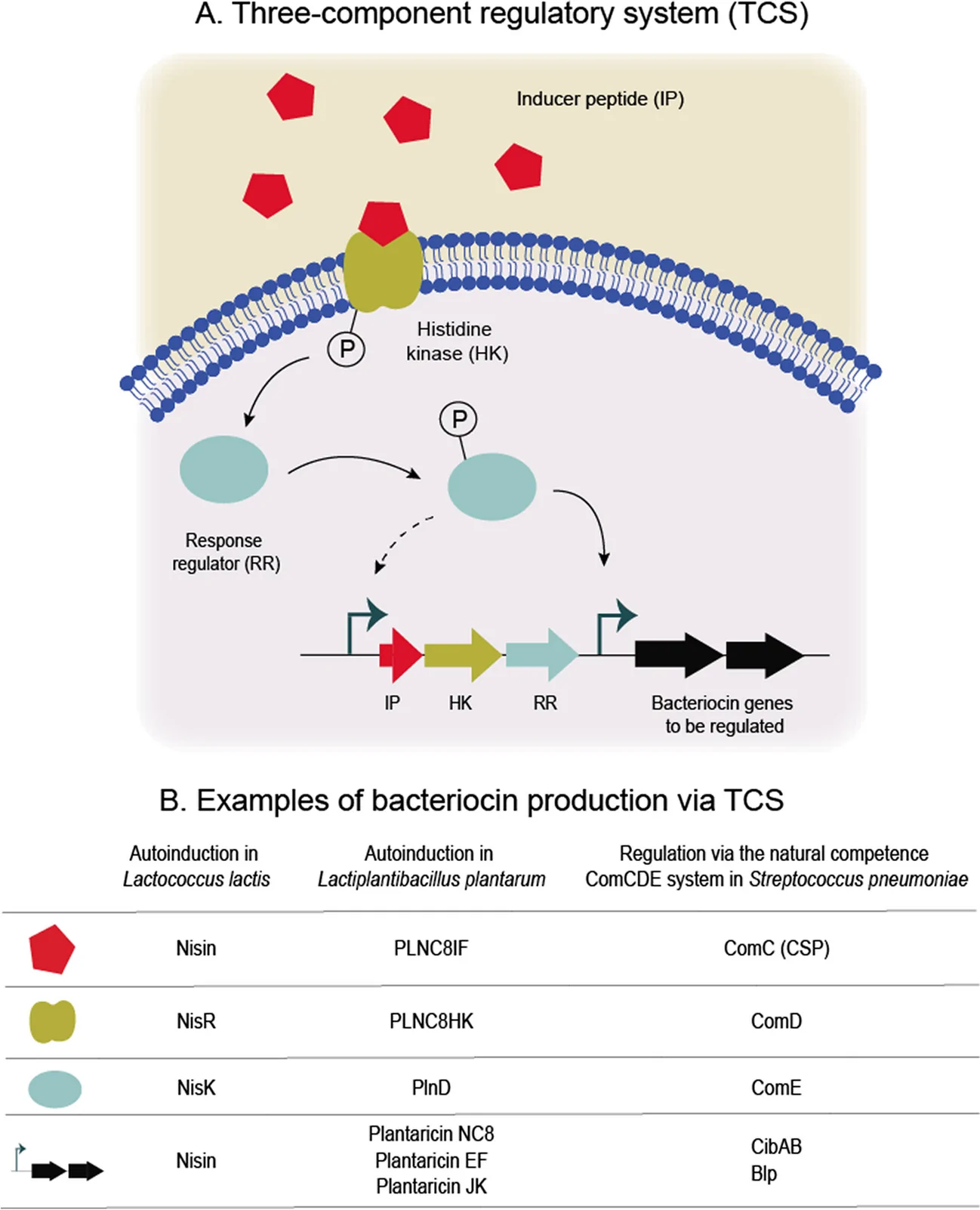
Regulation of bacteriocin production via three-component systems (TCS). Bacteriocin production can be regulated through the bacteriocin biosynthetic machinery by autoinduction or as part of natural competence systems. **A** In a typical three-component system a dedicated inducer peptide is produced in a cell density-dependent manner in response to environmental signals. When it reaches a certain threshold, the transmembrane histidine kinase sensor is autophosphorylated and transfers the phosphate group to the response regulator. The phosphorylated response regulator binds to target DNA elements activating the transcription and production of bacteriocin genes. **B** Examples of bacteriocin production via TCS: autoinduction of nisin production in *Lactococcus lactis* using nisin as autoinducer [112], autoinduction of plantaricin production in *Lactobacillus plantarum* NC8 through the pheromone PLNC8IF [49], and CibAB and Blp bacteriocin production in *Streptococcus pneumoniae* via the ComCDE natural competence system [113–115]

Bacteria have often evolved to express the bacteriocins only when they are needed and give benefits for the producer. Understanding the regulation of bacteriocins will thus also give important clues about their functional roles. Different regulatory mechanisms have been described and bacterial cells are able to regulate bacteriocin production in response to the growth phase, the physical state of the cell, and environmental factors such as nutrient scarcity, iron availability or osmolarity. The most well-known regulatory systems are quorum-sensing mechanisms governed by dedicated two- or three-component regulatory systems, allowing activation of bacteriocin production by autoinducers as a response to high cell densities and/or other environmental factors, relevant for instance in biofilm settings [105–107] (Fig. 2A). Such systems are discussed in detail below. In addition, bacteriocin production has been shown to be regulated by dedicated regulators [108], conserved sigma factors [109], or extracytoplasmic sigma factors [110]. A comprehensive overview of regulatory mechanisms controlling the synthesis of modified peptides was published recently [111].

In two- or three-component regulatory systems (Fig. 2A), the autoinducer may be the bacteriocins themselves (Fig. 2B). This is the case of the lantibiotics nisin, subtilin, or mersacidin, or the class II bacteriocin plantaricin A [112, 116–118]. In other cases, dedicated bacteriocin-like autoinducers without antimicrobial activity function to control bacteriocin production. Examples of the latter include the BlpC peptide of the streptococcal Blp-system, the PLNC8IF in *Lactiplantibacillus plantarum* [49] or SppIP, in *Latilactobacillus sakei*, respectively [119]. Interestingly, plantaricin A shows a bacteriocin or pheromone activity depending on its concentration adopting different modes of action [118]. On another aspect, environmental external stimuli can also affect bacteriocin expression. Low salt concentration and short times of exposure to gastric acid, simulating gastrointestinal conditions, stimulated bactofencin A promoter activity [120], antibiotics triggered Blp production in *Streptococcus pneumoniae* [113] and acetic acid stress increased bacteriocin production in *Lacticasibacillus paracasei* [121].

An intriguing regulatory and potentially functional link between bacteriocins and horizontal gene transfer is known from streptococci. In several streptococcal species, including *S*. *pneumoniae*, *Streptococcus mutans*, and *Streptococcus salivarius*, intertwined regulation between expression of bacteriocins and competence for natural transformation has been documented [107, 114, 122] (Fig. 2B). In these species, competent cells are able to take up naked DNA from their surroundings, and the co-regulation with bacteriocins suggests that bacteriocin-mediated killing and lysis of cells may contribute to rendering DNA from neighboring cells accessible for uptake [9]. Regulation of bacteriocin expression is intertwined with competence regulation in different ways [107, 122]. In *S. pneumoniae*, competence is regulated by quorum-sensing via the two-component system ComCDE, where a competence-stimulating peptide (CSP) encoded by ComC is produced and exported to bind to the histidine kinase ComD, which in turn phosphorylates and leads to transcriptional activation of the competent state via the response regulator ComE (see comprehensive reviews [123, 124]). The most straightforward example of co-regulation is that the phosphorylated response regulator ComE directly activates the transcription of the bacteriocin genes *cibABC*, a two-peptide bacteriocin found in *S. pneumoniae* [115]. A more intricate example involves crosstalk between the ComCDE system and a second related two-component system, BlpCRH, known to regulate the expression of bacteriocin-like peptides (Blp) [113, 114]. Here, CSP induces the production of Blp bacteriocins [113, 125] and the corresponding Blp-inducing peptide BlpC is dependent on the competence-expressed transporter for export [113].

Similar to the example above, the *Streptococcus gordonii* ComCDE competence system has also been shown to be linked to bacteriocin production [126]. Further examples of intertwined regulation are found in *S. salivarius* [127] where the ComRS regulatory system, another type of two-component system regulating competence in this species, also controls the production of bacteriocins [128]. Most likely, the same is true for bacteriocin production also in *Streptococcus thermophilus* [128]*.* Intriguingly, some species such as the dental caries associated *S. mutans* have both the ComCDE and ComRS competence regulatory systems [129]. In this species, the ComRS system is the main pathway for natural competence regulation while the ComCDE system is involved together with the VicRK signal transduction system in the regulation of the bacteriocins known as mutacins IV, V and VI [130].

The crosstalk and overlaps described here point to a common evolutionary origin of the regulation of bacteriocins and competence in these species [131], highlighting the potential relevance of bacteriocins in the evolution of natural competence. These intricate regulatory links may be a way for bacteriocins to facilitate evolution; bacteriocin-mediated lysis of cells releases DNA to the environment which may be taken up by natural transformation allowing the bacteriocin-producing cells to acquire new features. It remains to be determined how functionally relevant this feature is in nature and whether the coupling of competence and bacteriocin production observed in streptococci is a typical tactic to assist competent, bacteriocin-producing bacteria in other phylogenetic groups to acquire new traits.

## Broad and Narrow Spectrum Antimicrobials: Do They Have Different Roles?

Bacteriocins and antibiotics are both part of the bacterial antimicrobial arsenal. Although exceptions exist, bacteriocins generally show a narrower antimicrobial spectrum, most often targeting only bacteria closely related to the bacteriocin producer. Canonical antibiotics, on the other hand, have a wider spectrum of activity and even if their spectrum is restricted, they do not show preferential effect for strains related to their producer. As a result, it has been suggested that bacteriocins have a more limited impact on the surrounding microbiota, causing less disruption to the overall microbial community [132, 133]. Palmer and Foster developed an evolutionary model to explain why bacteria produce both broad-spectrum antibiotics and narrower-spectrum bacteriocins. These models suggest that narrow-spectrum bacteriocins are produced to eliminate relevant competitive threats efficiently while conserving resources, while broader-spectrum antimicrobials are produced when bacterial populations are abundant, aiming to establish dominance by displacing the wider community [134]. Thus, bacteriocins might target direct competitors while protecting others in their cooperative network. One could hypothesize that the production of narrow-spectrum bacteriocins represents a strategy to increase or maintain a high population diversity while effectively eliminating the primary competitors.

This is indeed in line with some studies of the distribution of bacteriocins in different environments. Garcia-Garcera and Rocha studied the production of extracellular proteins by bacteria, including bacteriocins in relation to the habitat spatial structure and the community diversity [135]. Their findings revealed a higher frequency of genes encoding bacteriocins in bacteria from diverse communities, and spatially structured habitats were also found to promote bacteriocin production. Similarly, bacteriocins have been suggested to play a vital role in promoting strain diversity during the establishment of microbiota in the infant human gut [136]. In this metagenome-based study, larger strain diversity was associated with the presence of bacteriocin genes in infant gut samples. Future experimental studies should aim at deciphering how and to what extent bacteriocins affect strain diversity in different environments.

## Biofilms and the Dynamics of Bacteriocin-Producing Populations

The surroundings and physical architecture of the microbial habitats are likely to have a great influence on the role bacteriocins play in nature. Some bacteriocins may, for instance, be prone to degradation by proteases and/or physically interact with surfaces, thereby limiting their stability and diffusion in different environments [137–140]. Therefore, bacteriocin activity is also dependent on the physical distance from bacteriocin producers to the susceptible target cells.

Biofilms, which are organized communities of microorganisms encased in extracellular substances, are the most common form of microbial life in nature [141]. One of the hallmarks of biofilms is the high density of cells, which may facilitate efficient antimicrobial activity of the bacteriocins due to the proximity of cells, but also promote activation of bacteriocin production through quorum-sensing regulation [49] (see section on “Regulation of bacteriocin production”).

The role of bacteriocins in biofilms has been studied to some extent. Bacteriocins have been widely tested as anti-biofilm agents inhibiting pathogenic biofilms [142] and shown to reduce cell surface adhesion inhibiting the early stages of biofilm formation and even disrupting established biofilms at subinhibitory concentrations [143–145]. This highlights the potential of bacteriocins as biofilm modulators also in natural settings. Indeed, when a bacteriocin-producing and a bacteriocin-sensitive strain are grown together in liquid culture, the bacteriocin producer outcompetes the sensitive one [146]. However, when the same strains are used in biofilm-experiments, they may be able to coexist [146]. This observation is attributable to the spatial localization of the cells within biofilms and highlights the impact of physical architecture on bacterial interactions [147, 148]. Biofilms may have variable degree of spatial segregation, ranging from well-mixed to complete spatial segregation of cellular subpopulations. It has been argued that bacteriocin production will be most favorable in intermediately mixed biofilms with local competition between strains. This may confer an optimal density of producer strains to initiate an effective attack and target susceptible cells in the vicinity [149, 150]. Too large degree of mixing may preclude the bacteriocin-producing bacteria to initiate a potent attack on target cells, while too large degree of segregation would limit the physical interaction between bacteriocins and susceptible cells. In the latter situation, bacteriocin-sensitive cells may indeed coexist with bacteriocin-producing cells.

With respect to bacteriocin production, bacteria can, in a simplified manner, be classified as bacteriocin-producers, bacteriocin-sensitive or bacteriocin-resistant. The outcome of the different interactions between these groups of organisms and the influence of bacteriocins in these settings is dependent on the physical architecture of the microbial habitat. Colicin-producing *E*. *coli* have long served as a model to study complex bacteriocinogenic interactions in in vitro and in in vivo mouse studies [151–153]. In environments with spatial segregation, the coexistence of the three phenotypes (bacteriocin-producer, bacteriocin-sensitive, or bacteriocin-resistant) has been shown to follow a non-transitive competition model in which there is no dominant competitor but a balance in which each of the phenotypes can outcompete one of the competitors [151]. The bacteriocin-producer displaces bacteriocin-sensitive cells, the producers are outcompeted by the resistant cells, which do not have a metabolic burden to produce the toxin, and the resistant cells can also be outcompeted by sensitive cells, since the latter would grow faster. This model, referred to as the rock-paper-scissors cyclic model, enables coexistence and favors microbial diversity on a short time scale when there is spatial separation [154]. In well-mixed systems, such as culture flasks, this microbial balance seems to be lost after prolonged incubation, and the resistant strain displaces the bacteriocin-producing and the sensitive ones [151, 152]. Resistant cells avoid the metabolic cost of bacteriocin production while still benefitting from the producers killing sensitive cells.

However, stable cyclic interactions, such as in the rock-paper-scissors model, seem to be unlikely in the long term [155], and different authors have suggested that interactions within the range from purely transitive to purely non-transitive competition are more likely to occur [156, 157]. Interestingly, in a study where engineered *E*. *coli* strains producing colicins of different antimicrobial potencies and mechanisms of action (colicin E3, E7, and V) were cultured together, the strain producing the less potent bacteriocin of the three, colicin V in this setup, outcompeted the others [155]. Epidemiological models for bacteriocinogenic *S*. *pneumoniae* have shown that diverse bacteriocin profiles from different bacterial populations can also be maintained either by the rock-paper-scissors model or by a “competition-colonization trade-off” mechanism [158]. In the latter, the bacteriocin producer would outcompete its non-producer counterpart within the same bacterial population, but pay a fitness cost in a context of other bacteria that are not sensitive [158]. Pyocins from *P*. *aeruginosa* are also important factors in driving competitive interactions in biofilms of these species. While different R-pyocin producers were able to compete each other, R-pyocins deletion mutants were outcompeted by wild-type R-pyocins producers, and combinations of R mutans were able to coexist spatially separated [159].

In a study with pyocin-producing *P. aeruginosa* isolated from different house locations, the isolates were more likely to outcompete each other when they were at an intermediate genetic distance [160]. The authors suggest that isolates from close communities are more similar to each other as a result of co-evolution and exclusion of sensitive phenotypes, while more separated isolates are more diverse with different pyocin profiles [160]. On a similar setup with *Pseudomonas fluorescens* isolated from soil at different locations in a park, no relation was found between inhibition and genetic similarity but inhibition among isolates was more frequent within strains from overlapping areas pointing to an important role of bacteriocin in resource utilization and niche competition [161].

A growing body of research highlights the links between bacteriocin producers and biofilm dynamics. However, the extensive diversity of bacteriocins presents a challenge in generalizing models for these complex interactions. Further studies are thus necessary to attain a comprehensive understanding of the intricate bacteriocin-based interactions, encompassing the various types of bacteriocins and experimental setups. Additional aspects, such as phenotypic heterogeneity, which could play a crucial role in sustaining resource-intensive traits like bacteriocin production [162] should also be explored in these investigations. Addressing these factors will contribute to a more complete understanding of the complexities underlying bacteriocin dynamics and their impact on microbial communities.

## Mobilization of Bacteriocin Genes by Horizontal Gene Transfer

Horizontal gene transfer (HGT), including transformation via natural competence, conjugation, and transduction, can have significant ecological and evolutionary implications in bacterial communities by transferring genes that modify the competitiveness of the strains. HGT is indeed important for the spread of bacteriocin genes between bacteria. Bacteriocin genes are often encoded on mobile genetic elements such as plasmids, and integrative and conjugative elements which facilitate their transfer between bacterial species or strains by HGT. Such phenomena have been observed for a large number of species, including the gram-positive *S. mutans* [8], *S. salivarius* [163], *Enterococcus faecalis* [164], *Staphylococcus aureus* [165] or the gram-negatives *E. coli* [166], and *Salmonella* [167]. Likewise, bacteriophage-associated bacteriocins have been detected in *Bacillus subtilis* [168], *Klebsiella* [169], and *E. coli* [170].

Strains acquiring complete loci gain the ability to produce bacteriocins, which may be advantageous for competition in natural settings. However, such uptake may also incur a fitness cost [171]. Krauss et al. showed that *S. aureus* acquiring a plasmid encoding micrococcin P1, a bacteriocin targeting protein synthesis, resulted in immediate production of the bacteriocin [165]. At the same time, this caused a major metabolic burden on the cell, which, interestingly, could be alleviated by increased expression of the central metabolic enzyme, citrate synthase [165]. Whether similar phenomena are true for bacteriocins targeting the cell membranes remains to be determined, but such phenotypes may represent hurdles for the spread of bacteriocin loci by HGT in nature.

In the context of HGT, it is also interesting to note that numerous studies have identified bacteriocin gene clusters that lack one or several essential components of a complete biosynthetic cluster. Some examples of incomplete bacteriocin clusters include *Pseudomonas* strains that carry the immunity genes but not the corresponding M-type bacteriocin structural genes [172], cyanobacteria harboring separated modification or transportation genes [95], and lactobacilli with partial operons that only contain structural and/or immunity genes [93]. Furthermore, several incomplete bacteriocin clusters have been identified when searching for circular bacteriocins in public databases [173] and so-called lasso peptides in ruminal bacteria [174]. Such incomplete loci may be the result of gene rearrangements, gene loss, or that these partial loci have been acquired by HGT [95, 175]. One potential result of the presence of such loci is the emergence of bacteriocin cheater phenotypes that exploit bacteriocin production by other members in the community without paying the cost of producing the bacteriocin. Isolated bacteriocin immunity genes can be utilized as a protective mechanism conferring cross-immunity against different bacteriocins [96, 176], and such cheaters may thus have important consequences for the dynamics of bacterial populations.

It is also possible that these incomplete clusters are in fact involved in functional bacteriocin production processes. For example, a phenomenon known as coexpression networks has been observed for fungal secondary metabolites in *Aspergillus niger* [177] and for the production of prochlorosins by cyanobacteria [178]. In these networks, genes involved in the production of specific metabolites are found at different locations in the genome. In bacteriocins from gram-positive bacteria, it is known that translocation and processing can occur via the general *sec*-dependent pathway, when no dedicated ATP-binding cassette transporters are found in the bacteriocin biosynthetic cluster [69, 179–182]. Experimentally, it is rare that all the genes required for bacteriocin production or genes in the bacteriocin structural gene vicinity are verified for their function, and therefore it is possible that bacteriocin production involves different pathways and circuits that might be located elsewhere in the bacterial genome.

## Non-antimicrobial Effects of Bacteriocins on Target Cells

Bacteriocins may have dual actions. In addition to their antimicrobial effect, they may also act as regulatory molecules at sub-lethal concentrations, similarl to what has been reported for antibiotics and other antimicrobial peptides [183, 184]. In addition to bacteriocin production itself being regulated by quorum sensing systems, sub-inhibitory concentrations of bacteriocins may induce a variety of cellular responses. Among bacteriocins produced by gram-negative bacteria, colicin M sub-inhibitory concentrations induced transcriptional changes in *E. coli* strains affecting genes involved in the cell envelope, osmotic stress, exopolysaccharide motility, and biofilm-associated genes [185]. Other examples include changes in the architecture *S. aureus* and *Staphylococcus epidermidis* biofilms after nisin application [186], and reduced levels of auto inducer 2 with low concentrations of subtilosin, a lantibiotic produced by *B. subtilis* [187].

In addition, it is known that proteins involved in regulatory pathways are directly influenced by bacteriocins. For instance, the leaderless LsbB family of bacteriocins targets the membrane-bound protease RseP [59] which serves as a key regulator for the stress response through the regulatory intramembrane proteolysis cascade across bacteria [57, 58]. Upon bacteriocin exposure, resistant enterococci acquire mutations in the site-2 protease *rseP* rendering them with an altered stress response capability [59]. Another example is streptomonomicin, a lasso peptide produced by *Streptomonospora alba* which influences regulation by WalR, a response regulator linked to cell wall metabolism [188]. *Bacillus anthracis* acquire streptomonomicin resistance by mutating *walR* rendering phenotypic defects including clumping of the cells, resistance to forming pellets, and long chains of cells. Moreover, the streptomonomicin-resistant strains showed upregulation of *liaI* and *liaH*, and downregulation of *ftsE* and *lytE* involved in the stress regulon and cell elongation, respectively [188]. Mutations in such regulatory systems typically render the cells more susceptible to various stressors, and the resistance to bacteriocins thus comes with a fitness cost.

To which extent the bacteriocins’ interactions within these systems can generate additional regulatory responses remains to be determined, but it could be speculated that bacteriocins (such as the leaderless bacteriocins targeting RseP [59]) act as communication modules at subinhibitory concentrations to target and modify diverse regulatory pathways in the population.

Interkingdom interactions are another aspect of the multifaceted nature of bacteriocins. In humans, bacteriocins have been found to interact at different levels with our immune system. For instance, plantaricin A is known to stimulate the synthesis of human-beta defensin 2 in keratinocytes [189], acidocin A and avicin A modulate cytokines and growth factors in human monocytes [190] and both nisin A and Z have immunomodulatory effects as reviewed elsewhere [191, 192]. In plants, bacteriocins from *Bacilli* spp. isolated from soils, for example, thuricin H7 [193, 194] and bacteriocin IH7 [195] can act as growth promotors.

## Conclusion and Outlook

Bacteriocins are ubiquitous among bacteria and extensive research has highlighted their versatility as competition molecules, population modulators, mobile elements, or as part of the cell communication pathways. However, studies on bacteriocins are scattered across the very different and diverse bacteriocin groups. Unraveling the full ecological significance of bacteriocins in different bacteriocin groups and their role in the microbial dialogue poses a challenge. Further multidisciplinary research is essential to gain a comprehensive understanding of bacteriocin circuits.

## Data Availability

No datasets were generated or analysed during the current study.
